# Identification of MicroRNAs and Their Target Genes Associated with Ovarian Development in Black Tiger Shrimp (*Penaeus monodon*) Using High-Throughput Sequencing

**DOI:** 10.1038/s41598-018-29597-y

**Published:** 2018-08-02

**Authors:** Chao Zhao, Sigang Fan, Lihua Qiu

**Affiliations:** 1 South China Sea Fisheries Research Institute, Chinese Academy of Fishery Sciences, Guangzhou, China; 2 Key Laboratory of Aquatic Genomics, Ministry of Agriculture, CAFS, Beijing, 100141 China; 3 Key Laboratory of South China Sea Fishery Resources Exploitation & Utilization, Ministry of Agriculture, Guangzhou, China

## Abstract

Plenty of evidence showing that microRNAs (miRNAs) post-transcriptionally regulate gene expression and are involved in a wide range of biological processes. However, the roles of miRNAs in ovarian development process remain largely unknown in shrimp. In the present study, high-throughput sequencing of small RNAs was performed to find specific miRNAs that are involved in ovarian development process in *Penaeus monodon*. Two small RNA libraries were constructed from undeveloped (UNDEV group) and developed (DEV group) ovarian tissues in *P. monodon*. In total, 43 differentially expressed miRNAs were identified between the two groups (*P* ≤ 0.05, |log_2_ ratio| ≥1), and their expression profiles were validated by qRT-PCR. In order to further clarify the functional roles of these differentially expressed miRNAs during ovarian development process, target gene prediction was performed. In total, 4,102 target genes of 43 miRNAs were predicted, then clustered by the Kyoto Encyclopedia of Genes and Genomes (KEGG) database; only four specific pathways related to ovarian development were obtained (*P* < 0.05). Dual-luciferase reporter assays and integrated expression analysis were also conducted to further clarify the interaction between the miRNAs and their target mRNAs. This study provides important information about the function of miRNAs involved in ovarian developmental stages in *P*. *monodon*.

## Introduction

The black tiger shrimp (*Penaeus monodon*) is one of the most commercially important penaeid species in Asia, especially in South China^1^. Although *P. monodon* has been reared for many years, aquaculture production and large-scale cultivation are still restricted by the supply of high-quality broodstock, especially in non-native countries such as China^2,3^. In recent years, unilateral eyestalk ablation has been applied broadly in the aquatic breeding industry to induce *P. monodon* ovarian maturation to generate large numbers of high-quality broodstocks^4–6^. However, this technique can lead to the death of the parent shrimp and lower spawning quality^4,7^. Therefore, the exploration of alternative approaches to unilateral eyestalk ablation is imperative. Although much effort has been expended in exploring such alternatives and some progress has been achieved, more effective methods are currently unavailable. Previous studies related to shrimp culture mainly focused on disease resistance mechanisms and culturing techniques^8–11^, and basic knowledge related to shrimp reproductive activity and molecular aspects of ovarian development remain scarce. In order to elucidate the molecular mechanism that regulates shrimp development and reproduction, it is important to study the regulatory mechanisms of ovarian development in this species.

Through the years, there have been disagreements among scholars, both domestic and overseas, concerning shrimp ovarian development. For example, Tan-Fermin and Pudadera believe that the process of ovarian process development should be divided into four stages: previtellogenic, vitellogenic, cortical rod, and spent^12^. However, Huang *et al*. divided the process of ovarian development process into six stages: I: primordial germ cell stage, II: chromatin nucleolus stage, III: perinucleolus stage, IV: yolky stage, V: cortical rod stage, and VI: spent stage^13^. In general, both the four-stage method and six-stage method can be condensed into two large stages, i.e., the undeveloped stage (UNDEV) and developed stage (DEV). The UNDEV ovary already has ovarian features and contains a large number of oogonial cells, but has yet to begin fast development, and no ovarian shadow can be observed by external inspection (i.e., combining I: primordial germ cell stage and II: chromatin nucleolus stage). The DEV ovary shows fast development, and the ovarian shadow begins to appear (i.e., combining III: perinucleolus stage, IV: yolky stage, V: cortical rod stage, and VI: spent stage). Furthermore, we can easily differentiate the two stages by simply observing the appearance of the shrimp, because the aforementioned ovarian shadow can be seen when the ovary begins to develop^13^. The main purpose of eyestalk ablation is to compel the UNDEV ovary to progress to DEV. Therefore, clarifying the difference in the molecular regulation mechanisms between shrimp UNDEV and DEV ovaries is very important to find an alternative technology to eyestalk ablation.

MicroRNAs (miRNAs) are small, non-coding RNA molecules, which have been implicated in the regulation of diverse cellular, physiological, and developmental processes in animals and plants^14,15^. miRNAs can regulate gene expression post-transcriptionally by binding to the mRNA of their target genes^16^. In recent years, intensive studies have been carried out on the miRNAs that mediate biological and metabolic processes in invertebrates, such as regulation of individual development^16–18^, abiotic stress response^15^, pathogen defense, and innate immune responses^19,20^; however, few studies have examined ovarian development, especially in shrimp. Although many studies have been carried out to uncover or expound genes related to ovarian development in shrimp, most are restricted to alterations in mRNA and protein expression patterns in different ovarian developmental stages^6,21,22^. Furthermore, expression profiling of the transcriptome and microRNAs between the UNDEV and DEV ovary remains largely unexplored in shrimp.

In the present work, we performed high-throughput sequencing of small RNA transcriptomes of ovarian tissues in UNDEV and DEV black tiger shrimps. Based on the analysis of small RNA transcriptomes conducted in this study, we identified known and novel miRNAs from the two different ovarian developmental stages. The results of qRT-PCR analysis, dual-luciferase reporter assays, and integrated analysis of miRNAs and their target mRNA expression profiles in the two different ovarian developmental stages allowed verification of miRNA and mRNA expression patterns and identification of miRNA-mRNA interaction pairs. Furthermore, the biological functions of the identified miRNAs were clarified by pathway analysis. This work provides genomic resources of miRNAs, which will be important for future studies in shrimp ovarian development, and provides data to support the development of alternative technologies to eyestalk ablation.

## Results

### Transcriptome sequencing data analysis, assembly, and annotation

In the present study, a normalized cDNA library from UNDEV and DEV ovaries of *P. monodon* was constructed and sequenced by the Illumina HiSeq. 2000 sequencing platform. A total of 87,407,166 sequencing reads were generated. After removing low quality reads and adapters, 85,559,880 (97.89%) clean reads were further retrieved, with an average GC content of 49.98%. The raw sequencing reads were submitted to the NCBI Short Read Archive under the accession number SRP132651. All the filtered short reads were further assembled *de novo* into 26,516 unigenes with an average length of 910 bp and N50 of 1,538 bp (Table 1). The size distribution of these unigenes was illustrated in Supplementary Information, Fig. S1. All unigene sequences were annotated by searching against Nr, SwissProt, EuKaryotic Orthologous Groups (KOG), and Kyoto Encyclopedia of Genes and Genomes (KEGG) databases, which retrieved 12,084 (45.57%); 10,493 (39.57%); 9,584 (36.14%); and 5,943 (22.41%) matches, respectively. This provided a final total of 12,147 (45.81%) annotated unigenes (Table 2).

**Table 1:** Summary of de novo assembly of *P. monodon* transcriptome.

Genes Num	GC percentage	N50	Max length	Min length	Average length	Total assembled bases
26516	44.76%	1538	14095	224	909.63	24119863

**Table 2:** The results of annotation on unigenes by different database.

Total Unigenes	Nr	Swissprot	KOG	Kegg	Annotation genes	Without annotation gene
26516	12084	10493	9584	5943	12147	14369

### High-throughput sequencing of small RNAs (sRNA-Seq)

**Figure 1:**
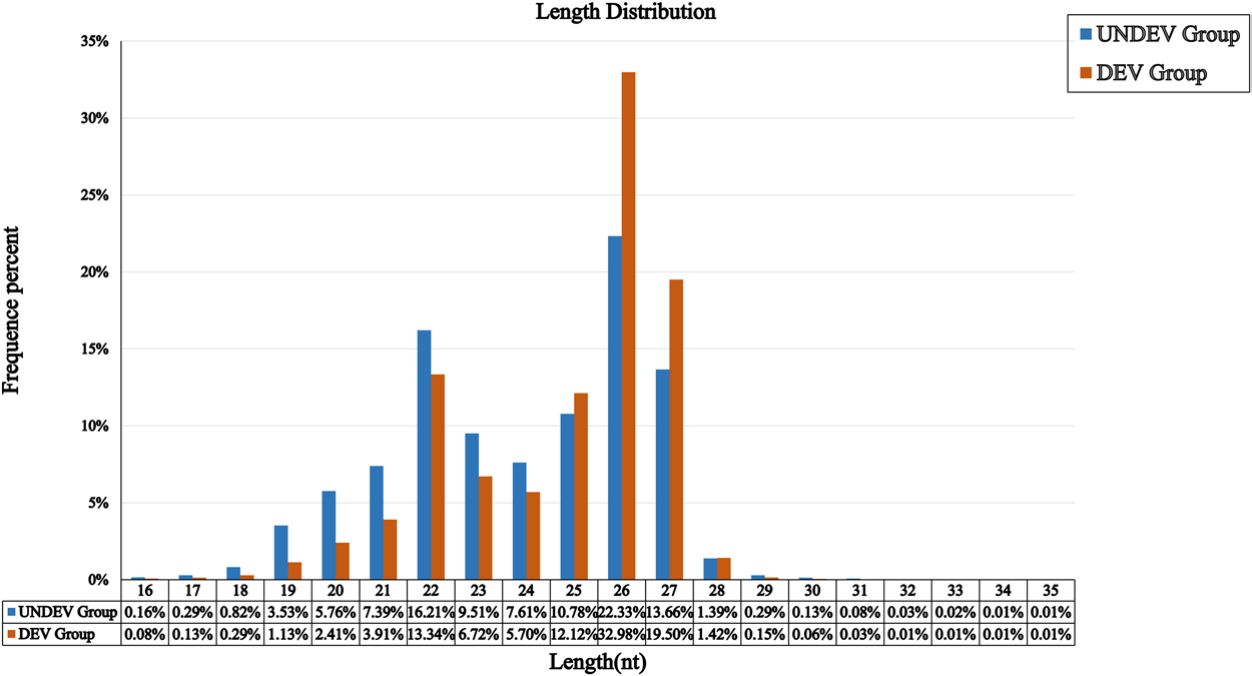
Length (nt) distribution of *P. monodon* sRNAs in UNDEV group and DEV group.

Two small RNA (sRNA) libraries were primarily constructed from UNDEV ovaries (UNDEV group) and DEV ovaries (DEV group) of *P. monodon*. In total, 15,214,167 and 15,850,922 raw reads were obtained from the UNDEV group and DEV group by high-throughput sRNA transcriptome sequencing, respectively (Table 3). After removing low quality reads and adapters, a total of 14,179,678 (98.34%) and 14,842,619 (98.80%) clean reads were retrieved, respectively. Deep sequencing results showed that sRNA from the two libraries shared a similar length distribution pattern. Twenty-six nt sRNAs were the most abundant (22.33% in UNDEV group and 32.98% in DEV group), followed by 22 nt (16.21%), 27 nt (13.66%), and 25 nt (10.78%) in the UNDEV group, and 27 nt (19.50%), 22 nt (13.34%), and 25 nt (12.12%) in the DEV group (Fig. 1). The clean reads can be divided into several categories (i.e., miRNAs, tRNAs, rRNAs, snoRNAs, snRNAs, and other sRNAs). As shown in Table 4, miRNAs represented the largest category in both the UNDEV and DEV groups.

**Table 3:** Summary of sRNA-Seq of ovaries in UNDEV group and DEV group.

Type	UNDEV Group		DEV Group	
	count	percent(%)	count	percent(%)
Total_Reads	15,214,167	100%	15,850,922	100%
High_Quality	14,418,326	94.7691%	15,023,564	94.7804%
3′adapter_null	41,993	0.2912%	33,476	0.2228%
Insert_null	32,508	0.2255%	56,656	0.3771%
5′adapter_contaminants	12,640	0.0877%	11,135	0.0741%
Smaller_than_18nt	150,402	1.0431%	79,137	0.5268%
PolyA	1,096	0.0076%	541	0.0036%
Clean_reads	14,179,687	98.3449%	14,842,619	98.7956%

**Table 4:** Classification of sRNAs of *P. monodon* in UNDEV group and DEV group.

Classification	UNDEV Group		DEV Group	
	The number of sRNAs	The abundance of sRNAs	The number of sRNAs	The abundance of sRNAs
snoRNA	103	463	61	277
rRNA	7,794	225,418	7,675	72,229
snRNA	1,447	9,881	792	2,907
tRNA	3,452	35,528	2,577	21,610
miRNA	19,384	999,054	10,984	823,493
Other	2,876,130	13,908,397	2,896,461	14,745,596
Total	2,888,926	14,179,687	2,907,566	14,842,619

### Identification of known miRNAs

After we compared the sRNA libraries to known animal miRNAs in miRBase with the National Center for Biotechnology Information Basic Local Alignment Search Tool (BLAST2GO), a total of 497 and 332 known miRNAs were identified in the UNDEV and DEV groups, respectively (Table 5). Among these known miRNAs, 225 were identified in both groups (Supplementary Table S1). Further comparisons of the expression levels of these miRNAs in the two groups indicated that miR-9-np was expressed at the highest level, followed by miR-9-5p, miR-100-5p, let-7-5p, and miR-125-5p in both groups (Supplementary Table S1).

**Table 5:** Classification of sRNAs of *P. monodon* in UNDEV group and DEV group.

Classification	UNDEV Group			DEV Group		
	miRNA Numbers	unique tag Numbers	Tag abundance	miRNA Numbers	unique tag Numbers	Tag abundance
Aligned known miRNA	497	19,384	999,054	332	10,894	823,493
Aligned novel miRNA	24	92	782	22	96	784

### Identification of novel miRNAs

To search for potentially novel miRNAs in the two groups, the precursors of several unannotated miRNAs were identified based on the *P. monodon* transcriptome. A total of 24 novel miRNAs were identified in the UNDEV group and 22 novel miRNAs were identified in the DEV group, (Supplementary Table S2). Among these novel miRNAs, 22 were identified in both groups (Supplementary Table S2).

### Identification of differentially expressed miRNAs between DEV and UNDEV groups

**Figure 2:**
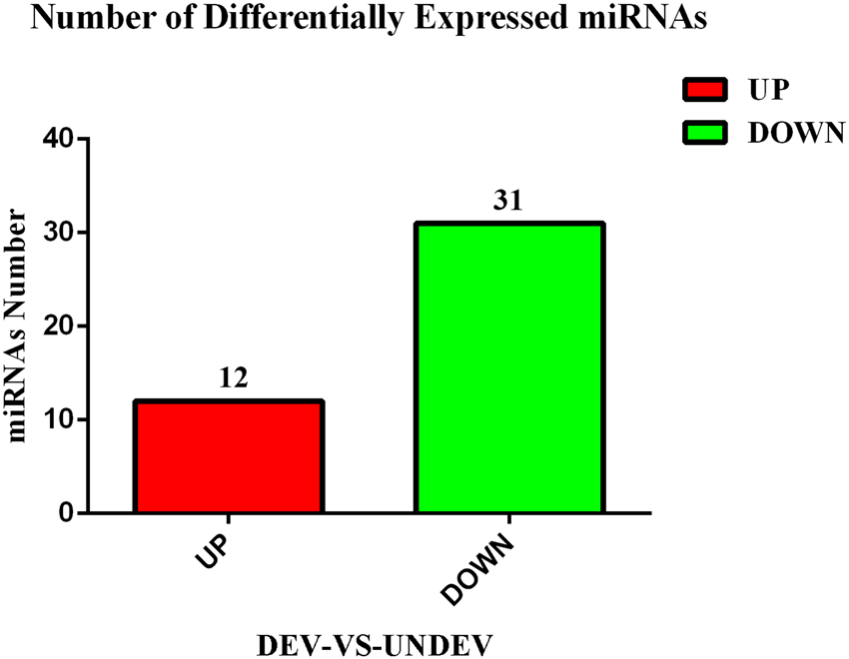
The numbers of differentially expressed miRNAs in the comparison between DEV and UNDEV groups. The red represented the number of miRNAs upregulated; the green represented the number of miRNAs downregulated.

For the purpose of identifying miRNAs that are relevant to ovarian development, the expression levels of all miRNAs were compared between the DEV and UNDEV groups. In total, 43 differentially expressed miRNAs were identified based on the statistical analysis of read counts (*P* ≤ 0.05, |log_2_ ratio| ≥1)^15^ (Supplementary Table S3). Among these miRNAs, 12 were upregulated, while 31 were downregulated (Fig. 2).

### Validation of differentially expressed miRNAs by qRT-PCR

**Figure 3:**
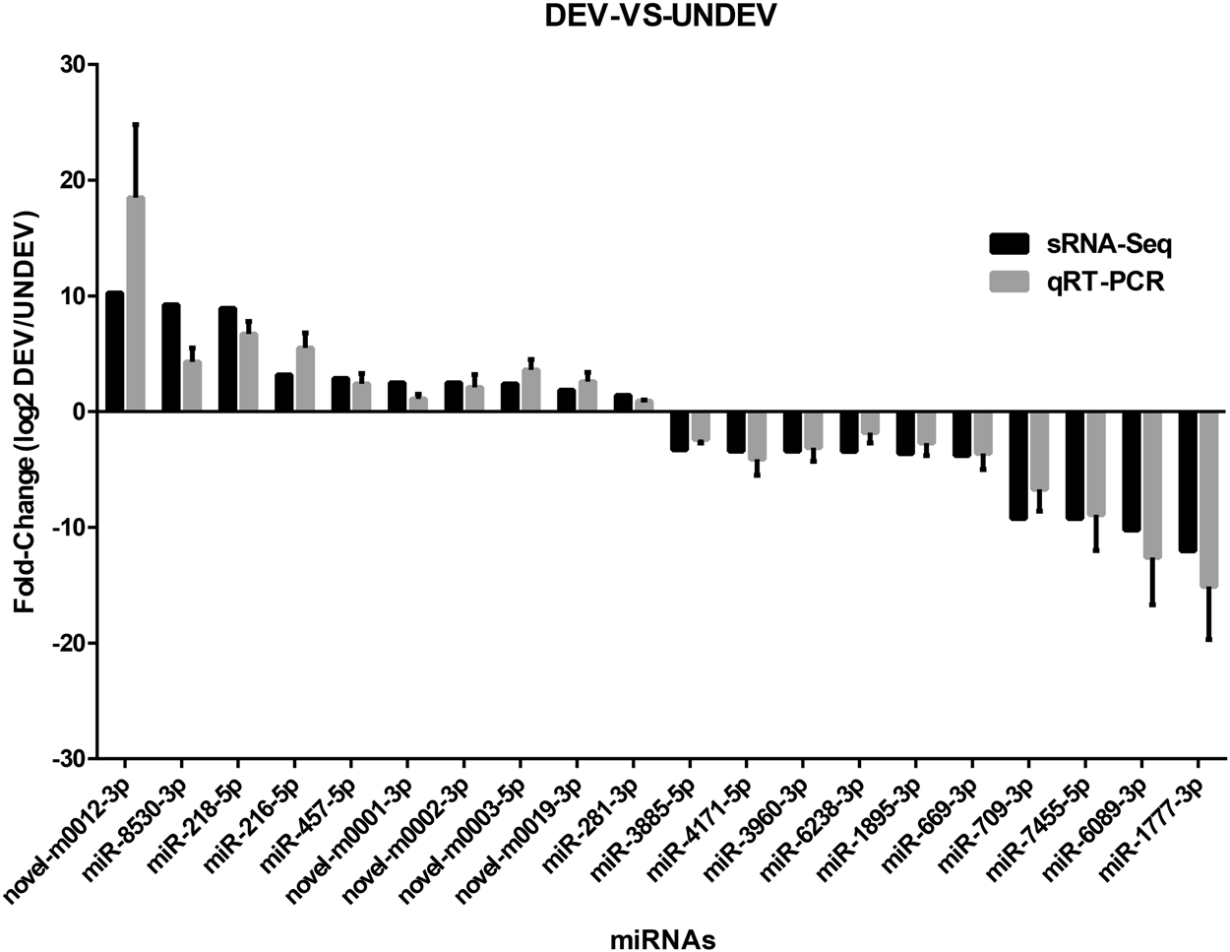
Differentially expressed miRNAs validated by qRT-PCR. Comparison between sRNA-Seq results and qRT-PCR validation results. X-axis shows miRNAs validated in this study; Y-axis shows Log2Ratio of expression of DEV versus UNDEV.

To verify the dynamic expression profile of the 43 differentially expressed miRNAs identified by high-throughput sequencing, 20 miRNAs were randomly chosen for qRT-PCR analysis (Fig. 3), using U6 snRNA as a reference gene. All of these miRNAs showed expression patterns that were consistent with the results from the high-throughput sequencing, indicating high reliability of the analysis.

### Target gene prediction and analysis

To better understand the functions of the 43 miRNAs, their putative target genes were predicted by TargetScan and miRanda based on the *P. monodon* transcriptome obtained above^23,24^. A total of 4,102 mRNAs were predicted to be target-regulated by the differentially expressed miRNAs (Supplementary Table S4). All 4,102 predicted mRNAs were further clustered by mapping the sequences into pathways in KEGG Mapper. Although 227 different pathways were found, only four specific pathways related to ovarian development were obtained (*P* < 0.05), specifically Oocyte meiosis (KO: 04114, *P* = 0.0006), Progesterone-mediated oocyte maturation (KO: 04914, *P* = 0.0042), Fatty acid biosynthesis (KO: 00061, *P* = 0.0116), and Cell cycle (KO: 04110, *P* = 0.0313). In total, 84 target genes were mapped to the four pathways (Supplementary Table S5); some of these genes had already been shown to be involved in the process of ovarian development in other organisms (Table 6). For example, the catalytic and regulatory subunits of mitogen-activated protein kinase kinase (*MAPKK*)^25^, extracellular signal-regulated kinase *(ERK)*^26,27^, fatty acid synthase (*FAS*)^28,29^, *cyclin A, cyclin B*^30–34^, and 14-3-3-like protein *(14-3-3-like)*^35,36^ were previously shown to be involved.

**Table 6:** The ovarian development related target genes and their corresponding miRNAs between DEV and UNDEV groups.

GeneID	Nr-annotation	Corresponding microRNAs
Unigene0006822	mitogen-activated protein kinase kinase (MAPKK)	miR-466-3p;novel-m0012-3p
Unigene0018463	extracellular signal-regulated kinase (ERK)	miR-1000-5p;miR-466-3p
Unigene0022872	Ras	mir-6489-3p
Unigene0009887	fatty acid synthase (FAS)	miR-2779-3p;miR-281-3p;miR-3741-3p;miR-6089-3p;miR-6493-3p;miR-669-3p;miR-709-3p;miR-8485-3p
Unigene0024651	PREDICTED: cell division cycle 7-related protein kinase-like (CDC7)	miR-8485-3p;
Unigene0023103	cyclin B	miR-466-3p;miR-750-3p;miR-6238-3p
Unigene0013859	cyclin A	miR-466-3p;miR-669-3p;miR-8485-3p
Unigene0018461	PREDICTED: DNA replication licensing factor mcm7-like (MCM7)	novel-m0019-3p
Unigene0013978	DNA replication licensing factor MCM4 (MCM4)	miR-6238-3p
Unigene0005881	Cell division cycle protein 27-like protein (CDC27)	miR-466-3p
Unigene0019921	14-3-3-like protein (14-3-3-like)	miR-1260-5p;miR-3741-3p;miR-3960-3p;miR-466-3p;miR-6238-3p;miR-669-3p

### Integrated expression analysis of miRNAs and their target mRNAs during ovarian development

**Figure 4:**
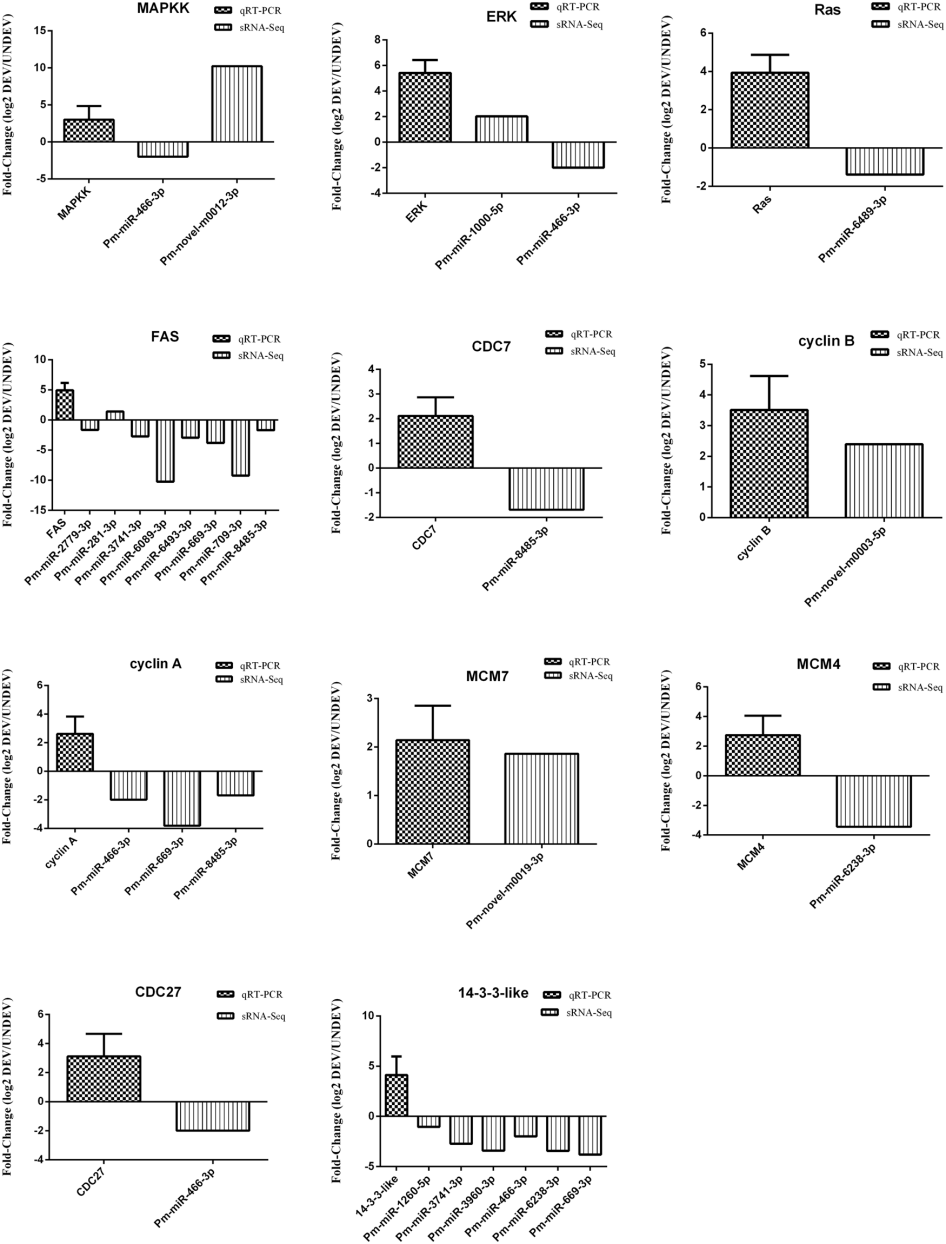
Integrated expression analysis of miRNAs and their target mRNAs by sRNA-Seq and qRT-PCR methods between DEV groups and UNDEV groups.

MicroRNAs can regulate target mRNAs either by RNA degradation or translational inhibition, depending on the complementarity between the miRNA and target mRNA. If expression profiling shows an inverse relationship between miRNA and mRNA levels, it means that one can regulate the other^15,37,38^. In this study, we compared the expression pattern of 11 selected mRNAs and their corresponding miRNAs between the DEV and UNDEV groups using the same samples (Fig. 4). Although the majority of mRNAs showed an inverse relationship with their miRNAs (Fig. 4C,E,G,I,J,K), some exceptions were detected (Fig. 4A,B,D,F,H). For example, the comparisons of *MAPKK* versus Pm-novel-m0012-3p, *ERK* versus Pm-miR-1000-5p/Pm-miR-466-3p, Fatty acid synthase (*FAS*) versus Pm-miR-281-3p, and minichromosome maintenance complex component 7 (*MCM7*) versus Pm-novel-m0019-3p showed a positive relationship.

### Validation of the interactions between miRNAs and their target mRNAs by dual-luciferase reporter assays

**Figure 5:**
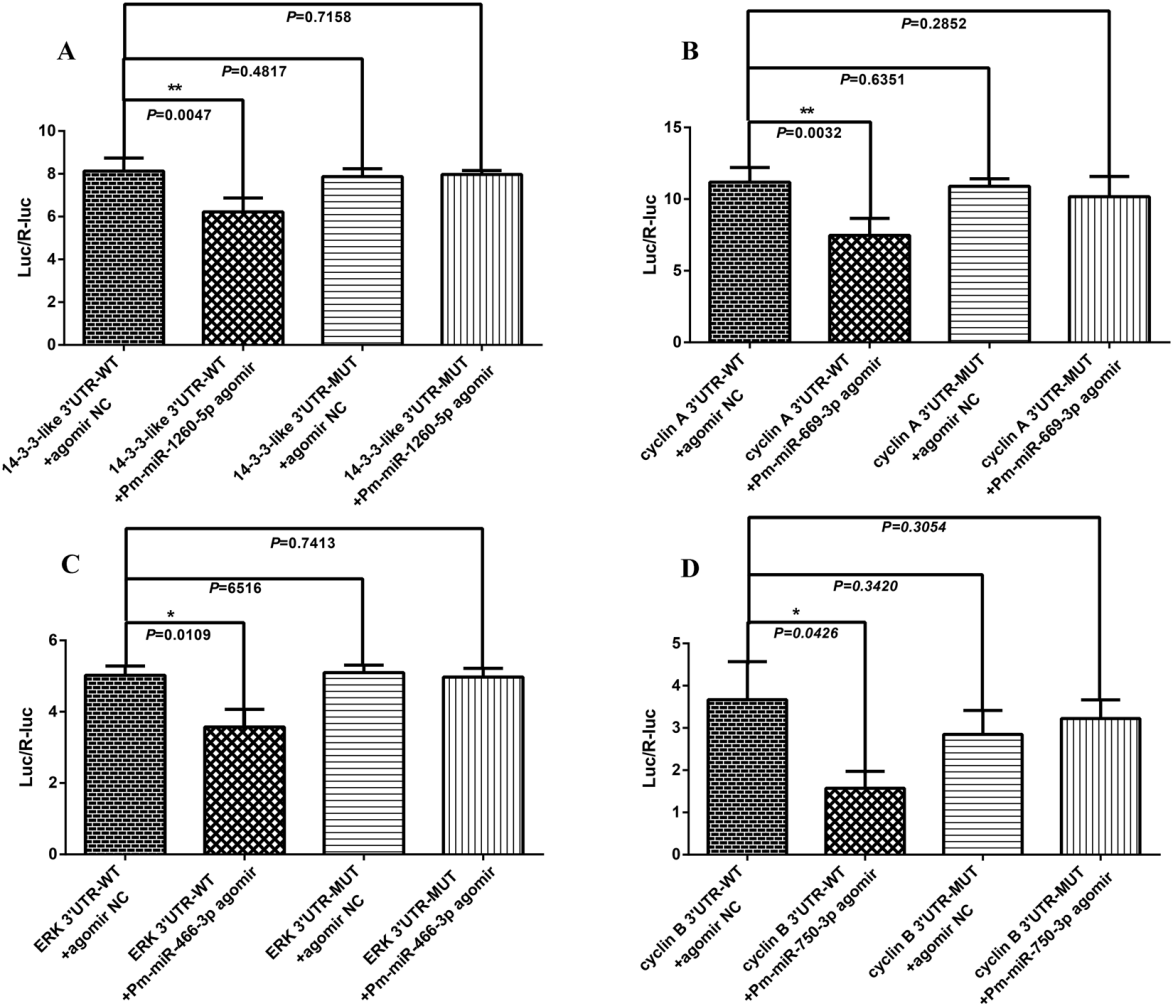
The sequence information about the binding and mutant sites of each miRNA at the 3′-UTR of the target genes. (**A**) the 3′-UTR of *14-3-3-like* and miR-1260-5p; (**B**) the 3′-UTR of *cyclin A* and miR-669-3p; (**C**) the 3′-UTR of *ERK* and miR-466-3p; (**D**) the 3′-UTR of *cyclin B* and miR-750-3p.

To further validate the interaction between the selected target mRNAs and their miRNAs, four miRNAs and their four target genes were randomly selected for dual-luciferase reporter assays. The sequence information regarding the binding and mutation sites of each miRNA at the 3′-untranslated region (UTR) of its target gene is shown in Fig. 5. After 48 h transfection, the luminescence ratio in each group was detected and a significant decrease was observed in the experimental groups (i.e., 3′-UTR-WT + miRNA agomir) compared with that of the control groups (i.e., 3′-UTR-MUT + miRNA agomir NC); no significant changes were observed in the other two groups (i.e., 3′-UTR-WT + miRNA agomir NC and 3′-UTR-MUT + miRNA agomir) (*P* < 0.05) (Fig. 5). As the results show, a 23.43% (*P* = 0.0047), 33.26% (*P* = 0.0032), 28.83% (*P* = 0.0109), and 57.14% (*P* = 0.0426) reduction in luciferase activity was observed in HEK293 cells transfected with the 14-3-3-like 3′UTR-WT + Pm-miR-1260-5p agomir (Fig. 5A), cyclin A 3′UTR-WT + Pm-miR-669-3p agomir (Fig. 5B), ERK 3′UTR-WT + Pm-miR-466-3p agomir (Fig. 5C), and cyclin B 3′UTR-WT + Pm-miR-750-3p agomir (Fig. 5D), respectively, relative to the corresponding control group. Moreover, the 3′UTR-WT + miRNA agomir NC and the 3′-UTR-MUT + miRNA agomir both showed no significant changes in luminescence compared to the corresponding control group values. Thus, we concluded that the miRNAs can specifically regulate their target mRNAs, and the predicted interactions between the mRNAs and miRNAs were reliable.

## Discussion

The black tiger shrimp (*P. monodon*) is a commercially important aquaculture species in South China and Southeast Asia^39,40^. At present, the numbers and quality of broodstock sourced from wild populations are in decline because of overfishing, causing difficulties for producers of farmed shrimp. Although eyestalk ablation can induce broodstock ovarian maturation in *P. monodon*, this process can result in reduced egg quality and death of spawners^40–42^. Therefore, a better knowledge of the molecular controls of the reproductive system of this shrimp would be very useful to help resolve these problems. In order to elucidate the molecular mechanisms of ovarian development, we decided to investigate miRNAs and their target genes that are involved in this process.

Although the roles of miRNAs in post-transcriptional regulation have been clarified in recent years, little progress has been made in non-model species, especially in shrimp. The advances in high-throughput sequencing technology provide unprecedented opportunities to efficiently characterize the sRNA transcriptome in *P. monodon*. In the present study, in order to study the roles that miRNAs play in ovarian development, we performed high-throughput sRNA transcriptome sequencing in UNDEV and DEV ovaries in *P. monodon*. Considering the difficulties of sampling the entire ovarian developmental stages of *P. monodon* under natural conditions, the ovarian samples were divided into UNDEV and DEV groups, instead of using the traditional method of categorizing the ovarian developmental process. The results of sRNA-Seq provided a large amount of basic data related to ovarian development, and to better analyze these sequencing data, transcriptome sequencing was also conducted in this study. Although 43 differentially expressed miRNAs and their corresponding 4,102 target genes were identified between the UNDEV and DEV groups, only four specific pathways related to ovarian development were obtained after clustering with the KEGG database. These were Oocyte meiosis, Progesterone-mediated oocyte maturation, Fatty acid biosynthesis, and Cell cycle. In total, 84 target genes were mapped to these four pathways; some of these genes had already been shown to be involved in ovarian development in *P. monodon* or other organisms. To further confirm the reliability of sRNA-Seq, qRT-PCR was carried out. The expression patterns of 43 differentially expressed miRNAs were validated by qRT-PCR. All miRNAs that were selected for validation had expression patterns that corroborated the sRNA-Seq results, indicating high reliability of the high-throughput sequencing. Several previous studies have shown that miRNA-mRNA regulatory networks do not always produce predictable regulatory patterns^43,44^. The results of our expression analysis of miRNAs and their target mRNAs also showed mixed results, in which both negative and positive correlations were observed. For example, Pm-miR-466-3p, Pm-miR-6489-3p, Pm-miR-2779-3p, Pm-miR-8485-3p, and Pm-miR-466-3p levels negatively correlated with *PmMAPKK*, *PmRas*, *PmFAS*, *PmCDC7*, and *Pmcyclin A* levels, respectively, while Pm-novel-m0012-3p, Pm-miR-1000-5p, Pm-novel-m0003-5p, and Pm-novel-m0019-3p levels positively correlated with *PmMAPKK*, *PmERK*, *Pmcyclin B*, and *PmMCM7* levels, respectively. Generally, negative correlations between miRNAs and their target mRNAs are supportive of miRNA targeting, but positive correlation exceptions have also been noted^43,44^. Lately, some studies have shown that miRNA targeting has a negative or positive feedback regulation on the respective mRNAs^45,46^, which could account for the positive correlations in the present research. Our study showed that a single miRNA could target multiple mRNA, and vice versa, demonstrating a more intricate miRNA-mRNA regulatory mechanism than we had previously believed. It can be concluded that these miRNAs are responsible for both switching on/off and fine-tuning target mRNA expression during the process of ovarian development.

Ovarian development is a consecutive process, which involves activation or inhibition of many genes (e.g., *RAS*, *ERK*, *cyclin B*, and *CDC7*). Ras protein family members, which are ubiquitously expressed in all animal cells and organs, belong to a class of proteins called small GTPases, and are involved in transmitting signals within cells (i.e., cellular signal transduction)^47^. Ras activates several pathways, of which the mitogen-activated protein kinase/extracellular signal-regulated kinase (MAPK/ERK) pathway (also known as the Ras-Raf-MAPKK-ERK pathway) has been well-studied. This MAPK/ERK pathway plays a critical role in initiating oocyte maturation^48^. MAPKK is a kinase involved in cellular processes, including cell growth, proliferation, differentiation, and apoptosis^49,50^, which can activate ERK (also known as MAPK)^50,51^. ERK can induce mitosis and meiosis in eukaryotic cells by activation of maturation-promoting factor (MPF), which is a cdc2-cyclin B complex that initiates meiotic resumption of oocytes^52,53^. Furthermore, Visudtiphole *et al*. also demonstrated that cyclin A and cyclin B were both required during the final maturation of ovaries in *P. monodon*^30^. CDC (cell division cycle related protein kinase) proteins are enzymes, encoded by *CDC* genes, which are involved in the regulation of cell cycle and ovarian development in different species^54–57^. Han *et al*. suggested that CDC2 and cyclin B play essential roles in oogenesis and spermatogenesis in the mud crab (*Scylla paramamosain*)^58^, and Mahattanee *et al*. also concluded that CDC2 should play a functional role in the development of oocytes in *P. monodon*^57^. Typically, meiotic oocyte maturation is initially induced by MIH (maturation inducing hormone) and signaled by several pathways that lead to the activation of MPF by dephosphorylation of Thr14 and Tyr15 residues on CDC2 by CDC25 phosphatase^59–61^. In our present research, miRNAs were identified that target the ovarian development genes mentioned above. Although most of these miRNAs negatively regulate their target genes, several positive regulations also exist (e.g., both Pm-miR-1000-5p and Pm-miR-466-3p can increase the expression level of *PmERK*).

It is well known that lipids play an important role during the development of decapod crustaceans, not only as energy sources, but also as essential nutrients^62,63^. Some essential fatty acids (EFA) have also been shown to be of special significance for gonad maturation and brood quality^63,64^. Considering that FAS is a major lipogenic enzyme catalyzing the synthesis of long-chain saturated fatty acids from 2-carbon donors, the changes in expression of *FAS* could affect the process of ovarian development. In the present study, Pm-miR-2779-3p, Pm-miR-3741-3p, Pm-miR-6089-3p, Pm-miR-6493-3p, Pm-miR-669-3p, Pm-miR-709-3p, and Pm-miR-8485-3p were identified as negative regulators of *PmFAS*, while Pm-miR-281-3p upregulated *PmFAS*. 14-3-3 proteins belongs to a eukaryotic-specific protein family with a general role in signal transduction^65^. They have the ability to bind a multitude of functionally diverse signaling proteins, including kinases, phosphatases, and transmembrane receptors, which play roles in multiple signaling pathways, including those controlling metabolism, hormone signaling, cell division, etc^66,67^. Pm-miR-1260-5p, Pm-miR-3741-3p, Pm-miR-3960-3p, Pm-miR-466-3p, Pm-miR-6238-3p, and Pm-miR-669-3p were all identified as negative regulators of *Pm-14-3-3-like* mRNA.

## Conclusions

In conclusion, there were 43 miRNAs that were differentially expressed between the DEV and UNDEV groups in *P. monodon*, and a total of 4,102 mRNAs were predicted to be target-regulated. After KEGG Pathway enrichment analysis, only 84 target genes were selected as ovarian development candidate genes. Next, integrated expression analysis and dual-luciferase reporter assays were adopted to better clarify the interaction between the miRNAs and their target genes. The present study improves our understanding of miRNAs and mRNA regulatory mechanisms with respect to ovarian development. Further studies are required to explain the biological functions of miRNAs and their targeted mRNAs, and more detailed work is required to understand fully the mechanisms of miRNAs during ovarian development in *P. monodon*.

## Materials and Methods

### Ethics statement

The collection and handling of the animals in the study was approved by the Chinese Academy of Fishery Sciences (Beijing, China)’ animal care and use committee, and all experimental animal protocols were carried out in accordance with the guidelines for the care and use of laboratory animals at the Chinese Academy of Fishery Sciences (Beijing, China).

### Sample preparation and RNA extraction

**Figure 6:**
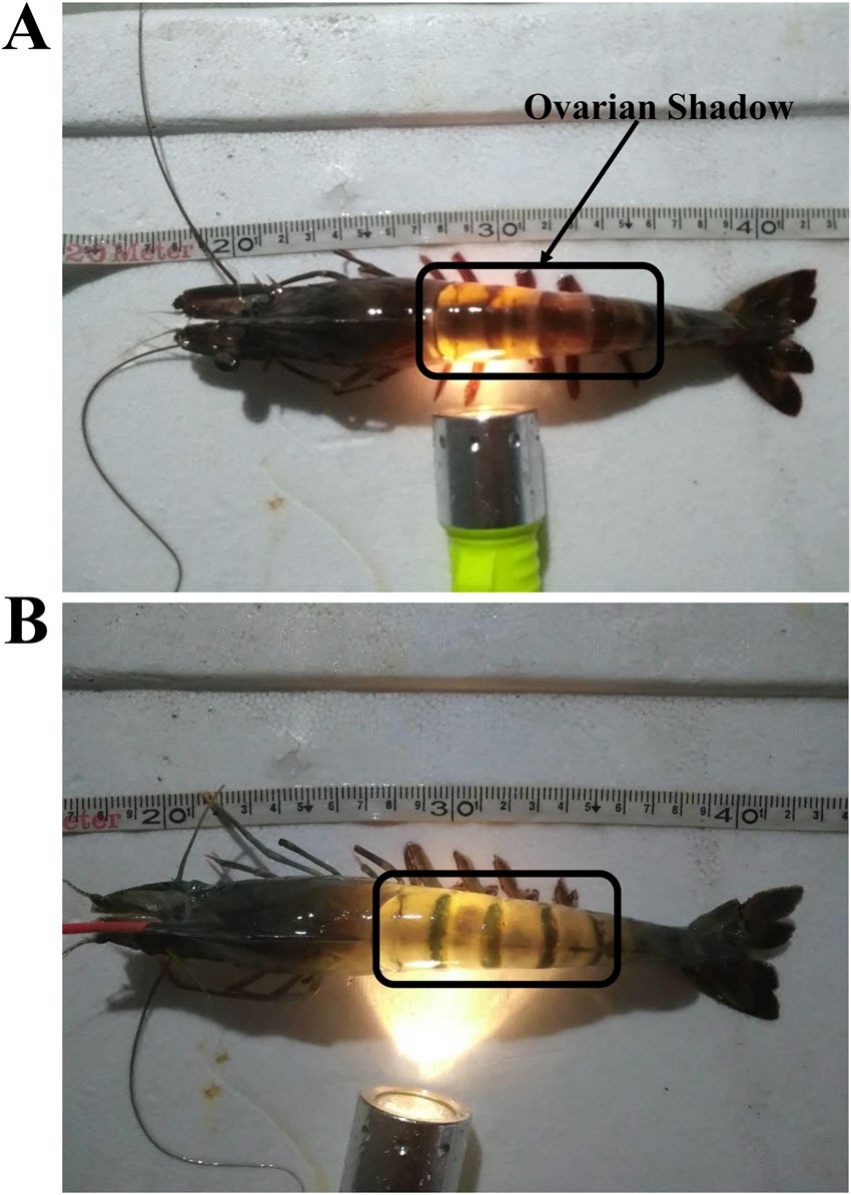
The interaction between miRNAs and their target mRNAs was validated by dual-luciferase reporter assays. (**A**) the interaction of *14-3-3-like* and miR-1260-5p; (**B**) the interaction of *cyclin A* and miR-669-3p; (**C**) the interaction of *ERK* and miR-466-3p; (**D**) the interaction of *cyclin B* and miR-750-3p.

The adult female black tiger shrimp (100–210 g) used in this study were purchased directly from the Sanya local market in Hainan Province in China. Because these shrimp were obtained from wild populations, it is impossible to know their ages accurately. Individuals were initially separated into UNDEV (Fig. 6B) and DEV (Fig. 6A) groups by observation of the ovary shadow on their backs. After separation, the shrimp were maintained at 25 ± 1 °C in two tanks containing aerated filtered seawater (31 ppt), with 50% of the water renewed daily. After acclimatization for 3 days, the shrimp were used for experiments. The shrimp were fed with commercial diets during acclimation until 24 h before treatment. Three shrimp from each group were dissected and the ovaries were collected immediately and stored in liquid nitrogen.

Total RNA was extracted from six tissue samples separately with RNAiso reagent (TaKaRa, Japan) according to the manufacturer’s instructions. RNA degradation and contamination were assessed initially by 1% agarose gel electrophoresis. RNA concentration and integrity were then assessed with the RNA Nano 6000 Assay Kit of the Bioanalyzer 2100 system (Agilent Technologies, CA, USA). RNA purity was verified using a Kaiao Photometer Spectrophotometer K5500 (Kaiao, Beijing, China). Total RNA from three tissues (3 µg each) from the UNDEV groups and three tissues (3 µg each) from the DEV groups were pooled, respectively.

### Transcriptome reference sequencing

Because no genomic sequences specific to *P. monodon* were available on public databases, we first performed *de novo* transcriptome sequencing and assembly. Three micrograms pooled RNA was used for mRNA library construction using the Illumina TruSeq RNA Sample Preparation Kit (Illumina) following the manufacturer’s recommendations. Briefly, mRNA was purified from 3 μg pooled RNA by using oligo d(T)-magnetic beads. After first and second strand cDNA synthesis, DNA fragments were blunted, adenylated at their 3′ends, and then ligated with Illumina phycoerythrin (PE) adapter oligonucleotides for hybridization. Then, cDNA fragments with lengths >200 bp were purified with the AMPure XP system (Beckman), and those ligated with adapters on both ends were selectively enriched using Illumina PCR Primer Cocktail in a 10-cycle PCR reaction. The products were purified again by the AMPure XP system and quantified using the Agilent 2100 bioanalyzer. Subsequently, a cluster of index-coded samples was generated using the TruSeq PE Cluster Kit v3-cBot-HS (Illumina) and sequenced on an Illumina Hiseq. 2000 platform. Finally, 100-bp paired-end reads were generated. After removing reads with adapters, reads containing ‘N’ (>10%), low quality reads (sQ ≤ 5), and redundant reads, the remaining clean reads were assembled using the TRINITY method^68^; redundant contigs were then screened by CAP3^69^. Finally, the unigenes were searched against the Nr database (NCBI non-redundant protein sequences) by Blast2GO^70^, and the orthologs were used as the reference sequences. All cDNA data series were submitted to the NCBI Sequence Read Archive (SRA) database with accession number SRP132651.

### Small RNA library construction and sequencing

According to the protocol of the Illumina TruSeq Small RNA Sample Preparation Kit (Illumina), 3 μg pooled RNA was used for sRNA library construction. In brief, RNA bands at ~20–30 bp were separated and purified by 6% Tris/Borate/EDTA (TBE) polyacrylamide gel electrophoresis (PAGE) and subsequently bound to 3′and 5′end adapters in two separate steps, followed by PAGE purification. After first strand cDNA synthesis using random oligonucleotides and SuperScript™ II, DNA fragments ligated with adapters on both ends were selectively enriched using Illumina PCR Primer Cocktail in a 12-cycle PCR reaction. Products of 145–160 bp (with adaptors on both sides) were separated by PAGE and quantified with the Agilent 2100 bioanalyzer. Then, a cluster of index-coded samples was generated using the TruSeq SE Cluster Kit v3-cBot-HS (Illumina) and sequenced on the Illumina Hiseq. 2000 platform. Finally, 50 bp single-end reads were generated. All small RNA data were submitted to the SRA database with accession number SRP133526.

### Filtering sRNA reads and microRNAs identification

After the unclean reads (i.e., the adapters, low quality reads, reads containing ‘N’, and redundant reads) were removed, the clean unique reads were mapped onto the *P. monodon* transcriptome reference sequences that were obtained in the present study using the program, Bowtie^19,71^. The reads with high sequence quality and ranging from 15–30 nt in length were annotated by searching against the GenBank^72^ and Rfam^73^ databases. For identifying orthologs of known miRNAs, the ideal mapped reads were checked against the metazoan mature microRNA (miRNA) of the Sanger miRBase (Release 19)^74^. Then, the non-conserved unique reads were filtered against Rfam (http://rfam.sanger.ac.uk/)^75^ and RepeatMasker (http://www.repeatmasker.org/)^76^, followed by using the program Bowtie to screen the sequences originating from rRNA, tRNA, snRNA, snoRNA, and repetitive elements.

### Analysis of conserved and novel miRNAs

Either reads mapped to non-miRNA in Rfam (e.g., rRNAs, tRNAs, and snoRNAs, etc.) or *P. monodon* mRNAs, were extracted for further research. The remaining reads were compared against miRBase 20.0^77^ and the *P. monodon* transcriptome reference sequences that were obtained in the present study for conserved and novel miRNA identification; stem-loop structure prediction was also performed. Conserved miRNAs were identified by using clean reads mapped to mature miRNA and hairpin sequences in miRBase 20.0. The reads that did not match the miRBase database were identified as unannotated. The unannotated data sets were aligned with the *P. monodon* transcriptome reference sequences that were obtained in the present study to predict novel miRNAs, using miRDeep2 software^78^ with the prediction of secondary structure.

### Expression pattern analysis of miRNAs

The read counts of each identified miRNA were normalized to the total number of reads to compare miRNA expression patterns between the UNDEV and DEV groups in *P. monodon*. In the present study, both DESeq^79^ and EdgeR^80^ were adopted to examine the differential expression between the two groups. Both methods have type-I error control^81^. Compared to that of DESeq, EdgeR has a lower sensitivity in detecting differentially expressed transcripts, but DESeq^82^ can identify more false-positive transcripts. To increase the accuracy of the results, the best strategy is to adopt more than one method and then combine the results. Therefore, only differentially expressed miRNAs that were identified by both methods were considered for further analysis. The fold change in miRNAs was calculated as the ratio of read counts in the treatment group to the read counts in the control group followed by log_2_ transformation. The miRNAs with absolute values of log_2_ (fold change) ≥1.0, and *P* < 0.05 were considered differentially expressed.

### Validation of differentially expressed miRNAs and mRNAs using qRT-PCR

For validating the expression patterns of miRNAs, reverse transcription was performed using the miScript II RT Kit (QIAGEN), after the miRNAs were extracted and purified from UNDEV and DEV ovarian tissues using the High Pure miRNA Isolation Kit (Roche) according to the manufacturer’s instructions. The synthesis reaction was incubated for 60 min at 37 °C, and terminated by heating at 95 °C for 5 min to inactivate the enzyme. With U6 snRNA serving as the internal control^83^, qRT-PCR was performed using the miScript SYBR^®^ Green PCR Kit (QIAGEN). The reactions were carried out in a total volume of 25 μL containing 2.5 μL of diluted cDNA, 2.5 μL of each primer, and 12.5 μL SYBR^®^ Green PCR Master Mix, with the following cycling profile: 95 °C for 15 min for polymerase activation, followed by 45 cycles at 94 °C for 15 s, 55 °C for 30 s, and 70 °C for 30 s. Using specific forward primers and the provided miScript Universal primers (Supplementary Table S6) and universal reverse primers, twenty miRNA fragments were amplified. Each sample was processed in triplicate and was amplified with the Roche LightCycler^®^ 480 (Roche). All data were analyzed using the 2^−ΔΔCt^ method.

For validating the expression patterns of mRNAs, target gene specific primers (F and R, Supplementary Table S6) were used during qRT-PCR to detect the temporal expression of genes in the black tiger shrimp. The housekeeping gene, elongation factor-1 alpha (*EF-1α*) (GenBank: DQ021452.1), was selected as the internal control. The qRT-PCR amplifications were processed in triplicate and were conducted on the Roche LightCycler^®^ 480 (Roche) in a total volume of 10 μL containing 5 μL of 2 × SYBR^®^ Premix Ex Taq (TaKaRa, Dalian, China), 2 μL of the cDNA template, 0.8 μL of 10 mmol·L^−1^ of each forward and reverse primer, and 1.4 μL of Milli-Q^®^ water. The cycling protocol for qRT-PCR was 94 °C for 30 s and 40 cycles of 94 °C for 5 s, 60 °C for 30 s, and 72 °C for 30 s. Melt curve analysis of the PCR product was performed from 65 to 95 °C at the end of each run to ensure that the correct product was amplified. The relative expression of the target gene was calculated according to the 2^-ΔΔCt^ method and normalized to the corresponding level of EF-1α^84^. SPSS17.0 was used to compute the Ct mean and standard deviation between replicate samples, and one-way ANOVA was adopted for the analysis of significant differences.

### Validation of interaction between miRNAs and their target mRNAs by dual-luciferase reporter assays

**Figure 7:**
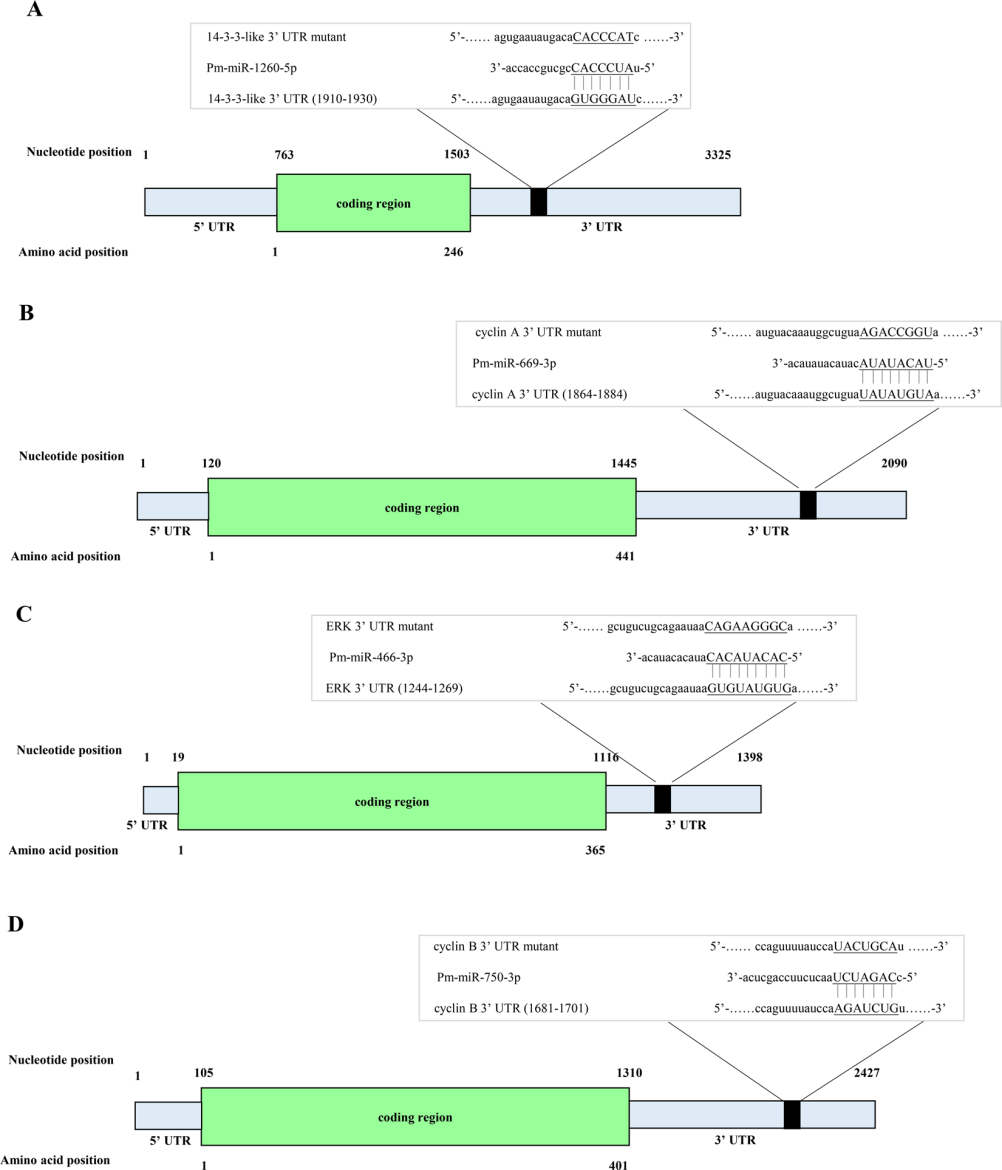
(**A**) Development female shrimp; (**B**) Undevelopment female shrimp.

The wild-type 3′-UTRs of 14-3-3-like (GenBank: AY903449.1), *cyclin A* (GenBank: EU707329.1), *ERK* (GenBank: GU324353.1) and *cyclin B* (GenBank: EU707332.1) mRNAs in *P. monodon*, containing the putative target sites of the miRNAs, were cloned into the pMIR-REPORT luciferase plasmid (Promega) between the *Mlu*I and *Hind*III restriction sites and were subsequently subjected to DNA sequencing for confirmation (Fig. 7). The primers used to clone the aforementioned 3′-UTRs are shown in Supplementary Table S6. The putative miRNA binding sites were mutated using a PCR approach and served as the mutant 3′-UTRs^19^. For the transfection experiment, HEK293T cells were seeded into a 96-well white TC plate in a total volume of 100 μL/well. Two solutions were prepared in each well as follows: the first solution contained 0.2 mg of pMIR-REPORT containing either the wild-type or mutated 3′-UTR and 0.01 mg of pRL-CMV constructs with 0.25 mL of transfection reagent. The second solution contained 100 nM miRNA mimics or 100 nM miRNA agomirs and 0.25 mL of transfection reagent. Twenty-five microliters of each solution was mixed together and incubated at room temperature for 20 min. Subsequently, the solutions were added to 50 μL of medium in each well. At 48-h post-transfection, the cells were collected and reporter activity determined using the Dual-Luciferase Reporter Assay System (E1910, Promega). The efficiency of each miRNA transfection was confirmed by fluorescence microscopy (BX53, Olympus). Luciferase activity was calculated based on the luciferase signal ratio between the two constructs, pMIR-REPORT and pRL-CMV, the latter being used to normalize the transfection efficiency among different samples. All of the experiments were performed in six replicates.

## Electronic supplementary material

Supplemantary information

Table S1

Table S2

Table S3

Table S4

Table S5

Table S6
