# Supplementary material for: Identification of MicroRNAs and Their Target Genes Associated with Ovarian Development in Black Tiger Shrimp (Penaeus monodon) Using High-Throughput Sequencing
Source: Sci Rep. 2018 Aug 2;8:11602. doi: 10.1038/s41598-018-29597-y (PMC6072753; doi:10.1038/s41598-018-29597-y)
Supplement: Supplementary file 1 — Supplemantary information [file 41598_2018_29597_MOESM1_ESM.pdf]

Identification of MicroRNAs and Their Target Genes Associated with Ovarian Development in Black Tiger Shrimp (*Penaeus monodon*) Using High-Throughput Sequencing

Chao Zhao [1, 3], Sigang Fan [1, 3], Lihua Qiu [1, 2, 3*]

[1]South China Sea Fisheries Research Institute, Chinese Academy of Fishery Sciences, Guangzhou, China.

[2]Key Laboratory of Aquatic Genomics, Ministry of Agriculture, CAFS, Beijing 100141.

[3]Key Laboratory of South China Sea Fishery Resources Exploitation & Utilization, Ministry of Agriculture, Guangzhou, China.

* Corresponding author E-mail: qiugroup_bio@outlook.com

Address of correspondence: Dr. Lihua Qiu

The South China Sea Fisheries Research Institute, Chinese Academy of Fishery Sciences

231 Xingangxi Road, Guangzhou 510300, P. R. China

Tel: +86-20-89108308, Fax: +86-20-84451442

Supplementary legends:

Figure S1. Length (nt) distribution of *P. monodon* Unigenes.

Table S1. Known miRNAs identified in the UNDEV group and DEV group, respectively.

Table S2. Novel miRNAs identified in the UNDEV group and DEV group, respectively.

Table S3. Significant differentially expressed miRNAs identified in the UNDEV group and DEV group, respectively.

Table S4. mRNAs that predicted to be target-regulated by the differentially expressed miRNAs.

Table S5. Identified target genes that mapped to Oocyte meiosis (KO: 04114, $P = 0.0006$), Progesterone-mediated oocyte maturation (KO: 04914, P=0.0042), Fatty acid biosynthesis (KO: 00061, $P = 0.0116$) and Cell cycle (KO: 04110, $P = 0.0313$) pathways.

Table S6. The primer sequences used for qRT-PCR and dual-luciferase reporter assay in this study.

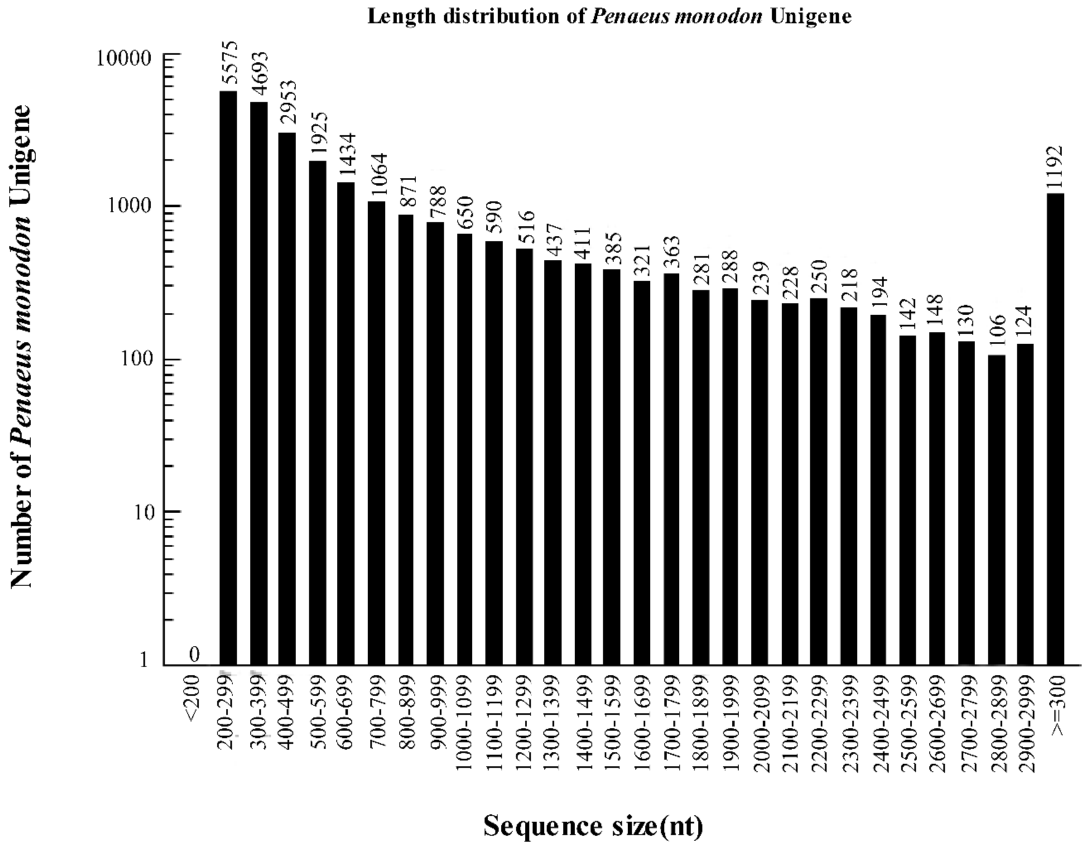

Figure S1. Length (nt) distribution of *P. monodon* Unigenes.
